# Bacterial extracellular vesicles and associated functional proteins in fermented dairy products with *Lacticaseibacillus paracasei*

**DOI:** 10.3389/fmicb.2023.1165202

**Published:** 2023-04-20

**Authors:** Gaspar Pérez Martínez, Lola Giner-Pérez, Keshia F. Castillo-Romero

**Affiliations:** ^1^Laboratory of Lactic Acid Bacteria and Probiotics, Department of Biotechnology, Instituto de Agroquímica y Tecnología de Alimentos (C.S.I.C.), Valencia, Spain; ^2^Laboratory of Neurobiology, Centro de Investigación Príncipe Felipe, Valencia, Spain; ^3^School of Engineering and Sciences, Tecnológico de Monterrey, Monterrey, Mexico

**Keywords:** *Lacticaseibacillus paracasei*, extracellular vesicles, fermented products, P40, P75, milk, 16S rDNA

## Abstract

Cells of all kingdoms produce extracellular vesicles (EVs); hence, they are present in most environments and body fluids. *Lacticaseibacillus paracasei* produces EVs that have attached biologically active proteins (P40 and P75). In this study, EV and functional proteins were found in five different commercial dairy-fermented products carrying *L. paracasei*. Strains present in those products were isolated, and with one exception, all produced small EVs (24–47 d.nm) carrying P40 and P75. In order to winnow bacterial EV from milk EV, products were subjected to centrifugal fractionation at 15,000 × *g* (15 K), 33,000 × *g* (33 K), and 100,000 × *g* (100 K). P75 was present in all supernatants and pellets, but P40 was only found in two products bound to the 15 and 33 K pellets, and 16S rDNA of *L. paracasei* could be amplified from all 100 K EVs, indicating the presence of *L. paracasei* EV. To investigate the interactions of bacterial EV and proteins with milk EV, *L. paracasei* BL23 EV was added to three commercial UHT milk products. Small-size vesicles (50–60 d.nm) similar to *L. paracasei* BL23 EV were found in samples from 100 K centrifugations, but intriguingly, P40 and P75 were bound to EV in 15 and 33 K pellets, containing bovine milk EV of larger size (200–300 d.nm). Sequencing 16S rDNA bands amplified from EV evidenced the presence of bacterial EVs of diverse origins in milk and fermented products. Furthermore, *L. paracasei* 16S rDNA could be amplified with species-specific primers from all samples, showing the presence of *L. paracasei* EV in all EV fractions (15, 33, and 100 K), suggesting that these bacterial EVs possibly aggregate and are co-isolated with EV from milk. P40 and P75 proteins would be interacting with specific populations of milk EV (15 and 33 K) because they were detected bound to them in fermented products and milk, and this possibly forced the sedimentation of part of *L. paracasei* EV at lower centrifugal forces. This study has solved technically complex problems and essential questions which will facilitate new research focusing on the molecular behavior of probiotics during fermentation and the mechanisms of action mediating the health benefits of fermented products.

## 1. Introduction

The discovery of extracellular vesicles (EVs) in biological fluids of all living organisms, including vegetables (Pérez-Bermúdez et al., 2017), prompted scientific interest on the likely role of EV in the deep interactions of food products with the host organism (Munir et al., 2020). Bovine and other ruminant's milk is rich in nutrients, immune and biologically active proteins, and generaly milk also contains abundant EV loaded with signaling proteins and miRNAs. Potential health benefits have been reported for milk EVs (Benmoussa et al., 2019a; García-Martínez et al., 2022); they are variable in size, proteomic profiles (Benmoussa et al., 2019b, 2020), and siRNAs content (Benmoussa et al., 2017). Nevertheless, industrial pasteurization and especially ultra-high temperature (UHT) treatment potentially alter the structure and composition of milk EV (Kleinjan et al., 2021). Fermented food products such as bread, fermented meats, olives, vegetables, and cheeses or fermented dairy products are essential for human nutrition. They require the presence and metabolic activity of conventional or unconventional microorganisms that provide pleasant organoleptic properties and extended shelf life, and in addition, they offer potential benefits for the consumer (host; Frias et al., 2016) and possibly contribute to enriching the gut microbiome (Milani et al., 2019). In particular, yogurt and fermented milk products are important references in healthy diets, which is supported by scientific evidence that showed that in addition to digestion and tolerance to lactose and improving the general gastrointestinal condition, yogurt and fermented milk products consumption reduced risks of breast and colon cancer, type 2 diabetes, weight control, and cardiovascular and bone health (Savaiano and Hutkins, 2021).

Bacteria also produce EVs with biological activity. EVs from gram-negative pathogenic bacteria showed virulence properties (Schwechheimer and Kuehn, 2015; Jan, 2017; Toyofuku et al., 2019) and pathogenic Firmicutes produce EVs with inflammatory effects, toxins, or virulence factors (Rivera et al., 2010; Gurung et al., 2011; Surve et al., 2016; Wagner et al., 2018). In contrast, EVs from probiotic bacteria have beneficial health properties, similar to the bacteria producing them, and these properties are related to their protein cargo, membrane-bound proteins, and lipoteichoic acids (LTA; Al-Nedawi et al., 2015; Domínguez Rubio et al., 2017; Grande et al., 2017; Bäuerl et al., 2020; Raftar et al., 2021; Fang et al., 2022). Probiotic species of the taxonomic group that includes *Lacticaseibacillus casei, Lacticaseibacillus paracasei*, and *Lacticaseibacillus rhamnosus* produce two characteristic and biologically active proteins, P40 and P75 (Bäuerl et al., 2019). These proteins have anti-inflammatory and tissue repair (anti-apoptotic) activities, and promote intestinal development in early life (Bäuerl et al., 2010; Yan et al., 2011, 2017; Wang et al., 2017; Lu et al., 2020). Furthermore, both proteins are externally bound to EVs (Bäuerl et al., 2020).

Given that *L. paracasei* (formerly *Lactobacillus casei*) is present in very popular and highly consumed fermented milk products, it was intriguing to know if commercial products had bacterial EVs and to what extent the functional proteins P40 and P75 were present and associated with bacterial vesicles. For this purpose, five commercial fermented milk products containing “*Lactobacillus casei*” were purchased. First *L. paracasei* strains were isolated and tested for the production of EV and the secretion of proteins P40 and P75. Unexpected results forced testing of the fate of these elements when EVs from the reference strain *L. paracasei* BL23 were mixed with sterilized milk. The results indicated that bacterial functional proteins—and possibly bacterial EV—combined with milk EVs and changed their sedimentation pattern.

## 2. Materials and methods

### 2.1. Bacterial culture, isolation, and identification

Isolation and regular culture of *Lacticaseibacillus* strains were carried out on Petri dishes with MRS medium and 1.8% agar or on liquid MRS, grown at 37°C under static conditions. For the isolation of *Lacticaseibacillus paracasei* strains from commercial products, fermented milk products of four different commercial brands that are labeled to contain probiotic strains of *Lactobacillus casei* (*Lacticaseibacillus paracasei*) were purchased. Product samples were arbitrarily encoded with letters A, Ca, Co, H, and Y. Serial dilutions of the five products were made on liquid MRS medium, and 100 μL of the 10^−5^, 10^−6^, and 10^−7^ surface inoculated dilutions on MRS agar plates was obtained in triplicate and incubated at 37°C for 48 h. Isolated colonies were purified and grown on MRS broth for 16S rDNA amplification and sequencing. An aliquot of this culture was preserved in 18% glycerol at −80°C.

For the identification of commercial isolates, the genomic DNA was isolated from a 5 mL MRS culture using the Real Pure SSS DNA Extraction kit (Real Durviz S.L., Valencia, Spain), following the manufacturer's instructions and described earlier (Bäuerl et al., 2019). Then, 16S rDNA was amplified with primers 27f (5'-AGAGTTTGATCCTGGCTCAG-3') and 558r (5'-GTATTCCGCGGCTG-3'), as described earlier (Marroki et al., 2011); next, amplicons (~500 bp) were purified with the Macherey–Nagel PCR Cleanup kit (Macherey-Nagel, CA, United States) and sequenced at Eurofins GATC Biotech (Eurofins Genomics, Germany). Sequences were compared with public databases through BLASTn.

Direct repeats in the gene encoding P75 (*cmu*B) from the isolated strains were sequenced together with BL23 as a reference. For this purpose, the chromosomal DNA of each of the strains was used as a template for the amplification of this region using two primers targeted 20 nt upstream, P75TR-for (5'-GCRAATGCTYCTAGCGCTGCTGC-3'), and 15 nt downstream, P75TR-rev (5'-GCCTGCCGTGACGGCGTAACAGG-3'), the satellite repeats. Then, sequences obtained were aligned to determine allele differences compared to those previously reported (Bäuerl et al., 2019).

### 2.2. Isolation of EV from bacteria and dairy samples

For EV isolation from dairy fermented products, samples were first neutralized with 0.75 M Tris pH 7.0 1:10 (v/v) and centrifuged at 4,000 × g to get rid of bacteria and other precipitates, as well as remaining fat. Then, supernatants were successively centrifuged to recover pellet fractions at 15,000 × *g*, 33,000 × *g*, and 100,000 × *g*, as indicated in the general procedure for dairy products (Benmoussa et al., 2017). For the isolation of milk EV, filtered (0.22 μm pore membrane) 2% sodium citrate was mixed (1:1 vol/vol) with 200 mL of commercial semi-skimmed UHT milk samples. Samples were successively centrifuged at 15,000 × *g* (15 K) for 20 min; the pellet was collected and the supernatant was centrifuged at 35,000 × *g* (33 K); and again, the pellets were collected and the supernatant was ultracentrifuged at 100,000 × *g* (100 K) for 2 h. Centrifugations at 15 and 33 K were carried out in a Beckman Coulter Avanti J-26 XPI centrifuge, and ultracentrifugation at 100 K in a Beckman Coulter Optima L-70 Ultracentrifuge rotor type Ti70 (Beckman Coulter, Indianapolis, Indiana, USA). The 100 K pellets were washed with 0.22 μm filtered PBS by centrifugation for 1 h at 100,000 × *g*, and in all cases, the temperature was fixed at 4°C. After each step, the pellets were resuspended in 1 mL of 0.22 μm filtered sterile phosphate-buffered saline (PBS).

Large amounts of *L. paracasei* BL23 EV were prepared from 400 mL of MRS supernatant. It was first centrifuged at 10,000 × *g* for 20 min at 4°C in 250 mL polypropylene bottles using a type 16.250 JLA rotor (Avanti J-26 XPI centrifuge, Beckman Coulter, Indianapolis, Indiana, USA). The supernatant was then filtered through a 0.45 μm membrane (Sarstedt, Nümbrecht, Germany) and concentrated 10-fold by tangential flow filtration (TFF), using a 100 KDa molecular weight cutoff (MWCO) polyether sulfone (PES) membrane (Vivaflow 50, Sartorius, Göttingen, Germany). The flow-through was ultracentrifuged at 100 K, as described previously.

### 2.3. Western blot

Immunological detection of P40 and P75 in EV samples was performed by Western blot analysis, as previously described (Bäuerl et al., 2019). In brief, the equivalent of 2 μg of EV protein was separated in 10% SDS-PAGE gels and electrotransferred to Hybond-ECL membranes (GE Healthcare, Boston, Massachusets, USA). Membranes were blocked in a 5% (w/v) non-fat dry milk solution in 50 mM Tris–HCl, pH 7.6 and 150 mM NaCl (TBS) containing 0.1% (v/v) Tween-20. The primary antibodies used were polyclonal rabbit anti-P40N and anti-P75N. Then, membranes were incubated with secondary goat anti-rabbit HRP-conjugated antibodies, and signals were detected using ECL advance chemiluminescent reagents, following the supplier's instructions (GE Healthcare, Boston, Massachusetts, USA; Bäuerl et al., 2019).

Western blotting was also implemented for the detection of *L. paracasei* LTA using the previously described protocol (Bäuerl et al., 2020). Using an SDS-free loading buffer and electrophoresis in a 17.5% polyacrylamide gel (SDS-free) was a key step in the procedure. Then, an anti-*Staphylococcus aureus* LTA mouse monoclonal antibody (G43J; Invitrogen, Waltham, Massachusetts, USA) was used as the primary antibody, and the rest of the procedure was followed as mentioned earlier.

### 2.4. Light scattering determination of EV size

The average size of isolated EV from BL23, from the newly isolated *L. paracasei* strains, from fermented dairy products, as well as milk preparations, was determined by DLS using a Zetasizer NanoZS (Malvern Panalytical Ltd, Malvern, Worcestershire, U.K.), with similar settings to those reported previously (Bäuerl et al., 2020) and by others (Grande et al., 2017). All samples required a previous filtration through 0.20 μm filters (Filtropur S, Sarstedt AG, Nümbrecht, Germany). Measurement conditions, replicates, cycles, and consumables were described earlier (Bäuerl et al., 2020). The refraction index of the samples was fixed at 1.330 and the viscosity was fixed at 1.0031 mPa/s. The equipment software calculated the average particle size using a third-order fitting autocorrelation function, as described by the manufacturer (Malvern Panalytical Ltd, Malvern, Worcestershire, U.K.).

### 2.5. Amplification and sequencing of bacterial DNA from EV

For PCR amplification from EV, DNA was isolated following the procedure described earlier (Yoo et al., 2016; Chang et al., 2021), with some modifications. In brief, product pellets after centrifugations at 15, 33, and 100 K were heated at 100°C for 20 min, and then centrifuged at 11,000 rpm in an Eppendorf centrifuge for 20 min. DNA in the supernatants was purified with the Macherey–Nagel PCR Clean-up kit (MACHEREY-NAGEL GmbH & Co., Promega Corporation, Madison, Wisconsin, USA). Then, 16S rDNA was amplified by PCR with the conditions cited earlier, with general purpose 16S bacterial primers 27f and 558r. New primers were designed for the specific amplification of 16S rDNA of strains belonging to the genogroup *Lacticaseibacillus casei/paracasei/rhamnosus/zeae* (Lcas215for- CCGCATGGTTCTTGGCTGAAAGAT; Lcas462rev- CGCCGACAACAGTTACTCTGCCGACCA). These amplicons were also sequenced to check the full 16S rDNA identity of *L. paracasei*.

## 3. Results

### 3.1. Characterization of *L. paracasei* strains from commercial products

A total of five different dairy-fermented products have been studied, of which two are commercialized by independent brands and the other three were distributors' white brands. All of them were similar liquid (not set) fermented milk products in small bottles (65–100 g per unit) carrying *Lactobacillus casei* (*Lacticaseibacillus paracasei*). Possibly, for logistics or marketing reasons, the right taxonomic assignment of these strains has not been updated on their labels. A total of four products (A, Ca, H, and Co) described in the label that, in addition to *L. casei* (*L. paracasei*), they contained yogurt starters, dairy ferments, or specifically described the presence of *Streptococcus thermophilus* and *Lactobacillus delbruekii* subs *bulgaricus*. Only one of the products (Y) mentioned the exclusive use of *L. casei* (*L. paracasei*). They all had low pHs between 3.80 and 4.65, and colony counts between 4.13 × 10^8^ and 13.2 × 10^8^ cfu/mL on MRS at 37°C (Supplementary Table 1). All strains were isolated and identified by sequencing the 500 nt 5' region of the 16S rDNA. Their sequences were almost identical and showed 99.6–100% identity to reference *L. paracasei* sequences, and then, they were given appropriate culture collection codes that corresponded to A, BL413; Ca, BL415; Co, BL416; H, BL417; and Y, BL418 (see also Supplementary Table 1). Furthermore, all five strains isolated were typed for the allele *cmu*B (encoding P75). Amplification and sequencing of the direct repeats containing the satellite region of this gene indicated that all strains had the same allele profile, identical to BL23 (*cmu*B1), with the exception of BL415 (FDP-Ca) which showed the deletion in allele *cmu*B3, following the pattern of strain *L. paracasei* TMV 1.1434 (Bäuerl et al., 2019; Supplementary Figure 1).

### 3.2. Isolation of EV and detection of associated P40 and P75 in commercial probiotics

All *L. paracasei* isolates were examined for the production of EV and associated P40 and P75 proteins. Supernatants of Lactobacilli grown on MRS were centrifuged at 15, 33, and 100 K successively, in order to follow the same procedure as with dairy products (see later). The presence and likely size of EV were determined by DLS in pellets recovered from the 100 K centrifugation. The amount and sizes of EV were variable between the strains. With the exception of BL416 (FDP-Co), all strains had two light scattering peaks corresponding to particles of 300–450 d.nm and much smaller size, between 24 and 67 d.nm [Table 1, which coincided with previously reported EV size reported for BL23 (Bäuerl et al., 2020)]. Larger particles detected on all samples could originate from EV aggregates (Table 1 and Supplementary Figure 2).

**Table 1:** The average particle size of EVs obtained for the lactobacilli isolated from different commercial products found in 100 K pellets and determined by DLS.

**Original product**	**EV**	**Pk 1 mean (d.nm; Sdev.)**	**Pk 2 mean (d.nm; Sdev.)**	**Pk 3 mean (d.nm; Sdev.)**
	BL23	309.9 (55.6)	47.1 (9.9)	–
A	BL413	241.7 (142.4)	43.2 (17.9)	–
Ca	BL415	491.9 (187.9)	27.6 (5.9)	–
Co	BL416	301.0 (59.3)	–	–
H	BL417	234.8 (136.0)	39.6 (26.1)	–
Y	BL418	425.5 (6.6)	94.2 (17.2)	24.8 (2.0)

**Figure 1:**
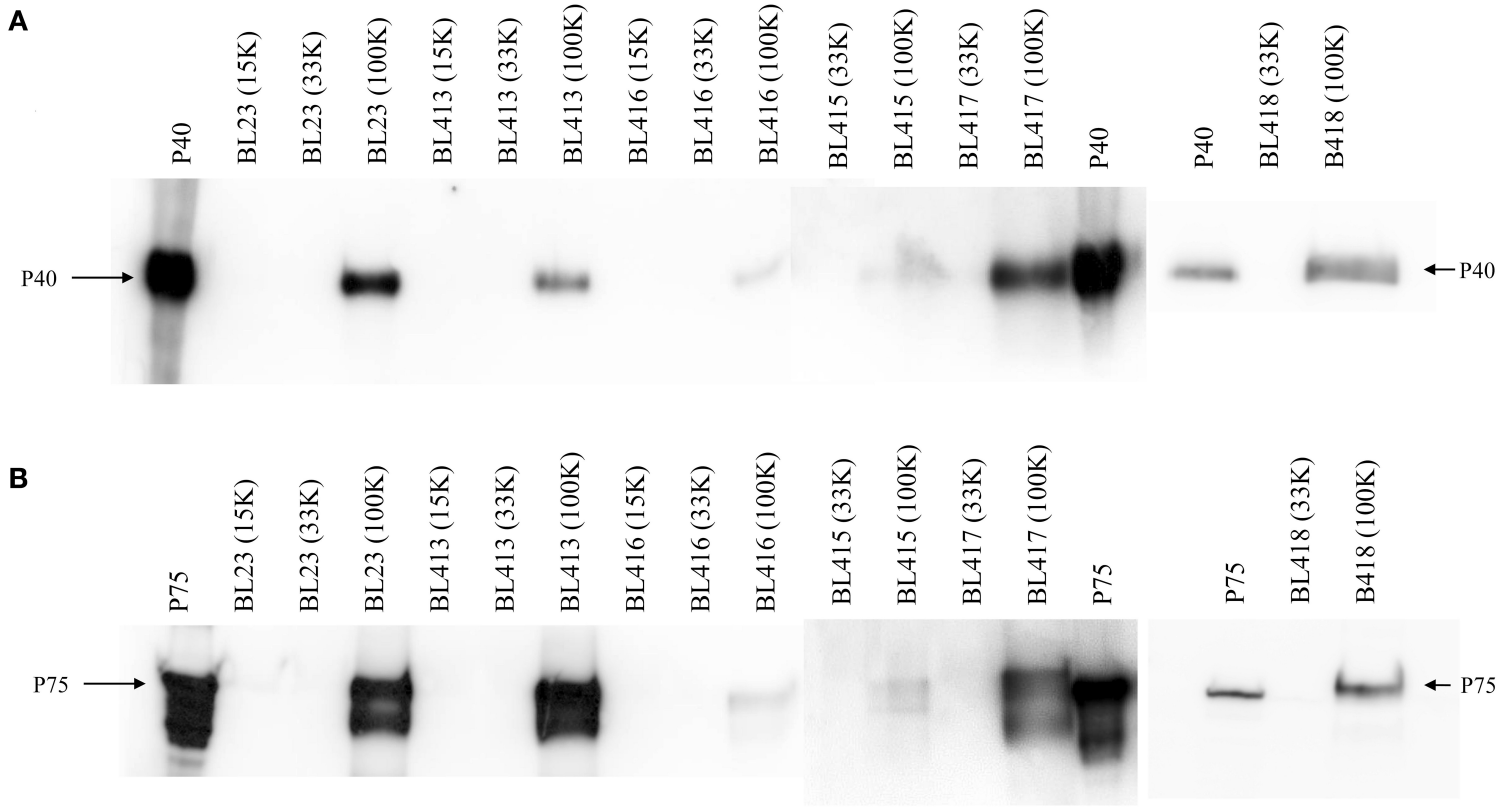
Western blot showing proteins P40 **(A)** and P75 **(B)** in EV isolated from the reference strain of *L. paracasei* BL23 and *L. paracasei* isolated from dairy fermented products (BL 413 from product A, BL 415 from product Ca, BL 416 from product Co, BL 417 from product H, and BL 418 from product Y). Control lines carry purified proteins P40 and P75 from BL23.

Then, the presence of P40 and P75 was monitored by Western blot in the EV obtained at 100 K and from pellets collected in previous 15 and 33 K centrifugations (Figure 1). All strains produced P40 and P75, and amounts detected in MRS had different intensities. They were found in the 100 K pellets, indicating that they were possibly bound to EV.

### 3.3. Identification of bacterial EV in fermented dairy products

EVs from fermented dairy products were isolated after successive centrifugations at 15 K, then the supernatant was centrifuged at 33 K, and this supernatant was centrifuged at 100 K. Pellets obtained after the different centrifugations were preserved at −80°C for further study. Due to the nature of those products, especially after 15 K centrifugations, pellets had a large volume and were difficult to dissolve in PBS, which made it difficult to analyze them by DLS. Only suspended vesicles from the 100 K pellets were stable and rendered good quality DLS signal intensity. The determination of the EV size showed that all the products had EVs of very similar size, yielding an average particle size of 147.26 d.nm (Sdev. 15.48; average z-value 120.95 d.nm, Sdev. 9.00; Supplementary Figure 3).

#### 3.3.1. Detection of EV-bound proteins P40 and P75

**Figure 2:**
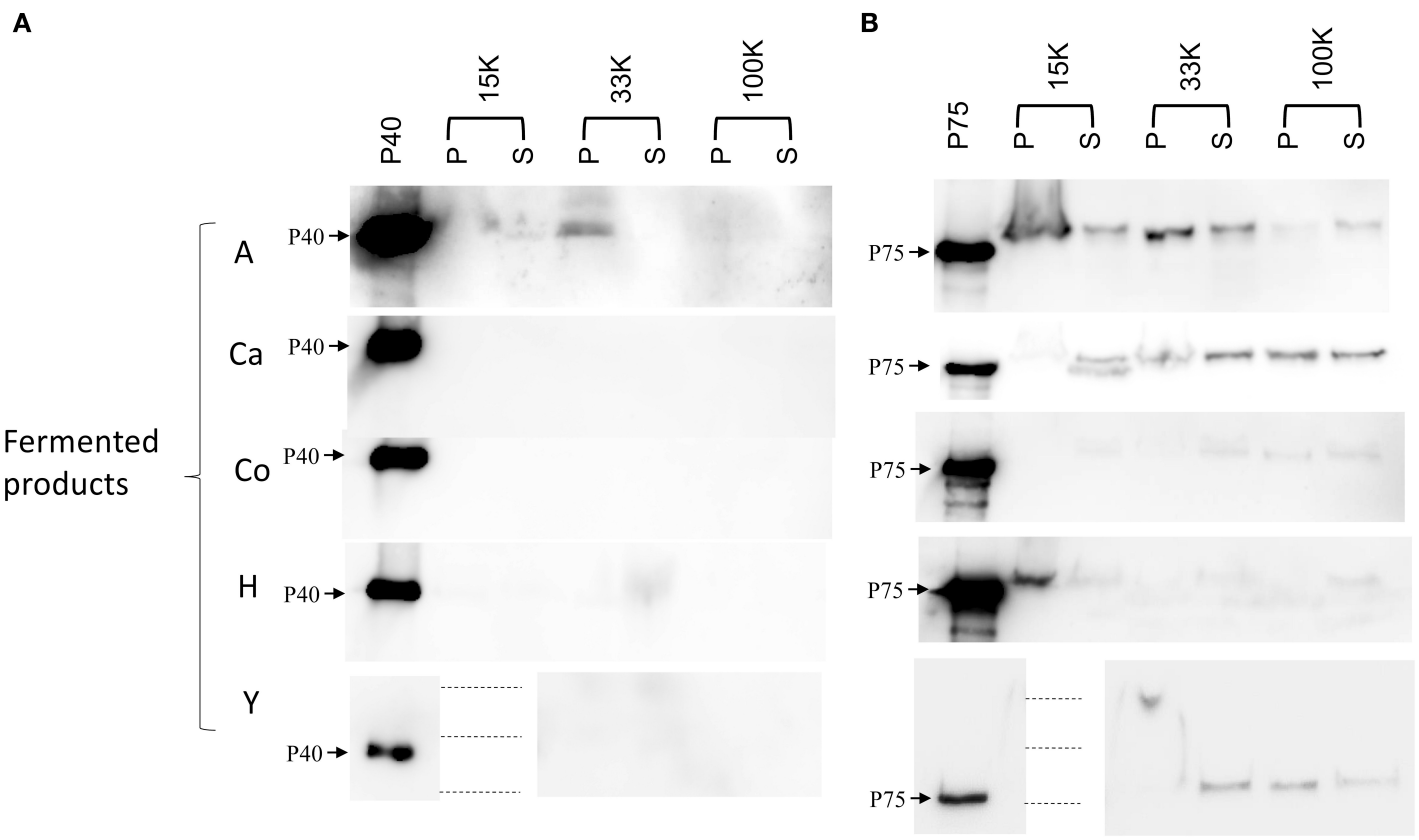
Detection of P40 and P75 in the pellets (P) and supernatants (S) of the sequential centrifugation (15, 33, and 100 K) of five fermented dairy products (A, Ca, Co, H, and Y). **(A)** Shows all samples detected with anti-P40 rabbit antibodies, and **(B)** shows samples detected with anti-P75 rabbit antibodies. Purified His-tag P40 and P75 were used as references for the corresponding blots. It was technically impossible to load on the PAGE samples of 15 K pellet of product Y; hence, both P and S samples of product Y were omitted, and the image has been split to align with the track annotations above.

In order to detect *L. paracasei* EV, pellets from 100 K ultracentrifugation were presumably carrying microbial EVs and the associated P40 and P75, but unexpectedly, P40 was not found in those samples. Then, Western blot was repeated for the inspection of P40 and P75 in all pellets and supernatants of fermented dairy products (15, 33, and 100 K; Figure 2). Due to the nature of the products, the 15 K pellets had a high volume and protein content, which saturated the nitrocellulose membrane blocking protein transfer in some samples. P40 could be clearly detected in product A (pellets of 15 and 33 K, and a very faint band in 100 K) as well as in product H (pellets of 33 K). In relation to P75, it was present in all the supernatants and pellets of all the products, normally yielding more intense bands in the corresponding pellet line. Products A, Ca, and Y had more intense bands than H and Co. In the case of product Y, it was particularly difficult to suspend the 15 K pellet in a small volume of buffer, and as a consequence, 15 K samples could not be analyzed.

#### 3.3.2. Confirmation of the presence of microbial EV in the samples

In order to double-check the presence of bacterial EV in the different pellets, Western blots under special LTA electrophoresis conditions were run (Bäuerl et al., 2020), but no signals could be detected possibly low amounts of EV and low sensitivity of anti-LTA antibodies (data not shown). Bacterial EVs have been reported to carry bacterial DNA cargo (Biller et al., 2014); hence, we proceeded to confirm the presence of bacterial EV by PCR amplification and sequencing of the 100 K pellets of all fermented dairy products. Initially, all 100 K samples were tested for the presence of bacterial DNA by sequencing amplicons obtained with general bacterial primers (Table 2). Sequencing of the amplicons indicated the presence of DNA from *Lactobacillus delbrueckii* subsp *bulgaricus* EV of two products (Co and H), *Streptococcus thermophilus* in product Ca, and *Pseudomonas gessardii* and *Pseudomonas aeruginosa*, respectively, in the 100 K EV fraction of products A and Y. Of note, with these universal 16S bacterial rDNA primers, all PCR reactions rendered a single high-intensity band at the expected size (~500 nt); however, quality of the sequences was low indicating that the amplicon had different DNA molecules. As a consequence, specific *L. paracasei* primers were designed to specifically detect EV of this probiotic species. Sequencing the corresponding amplicons showed that all the EVs analyzed contained *L. paracasei* 16S rDNA (Table 2).

**Table 2:** Sequences obtained after the amplification of 16S rDNA from EV in the 100 K pellets of the different fermented dairy products.

**EV from fermented dairy products**	**Universal bacterial 16S primers (27f-558r)**	***L. paracasei*** **specific 16S primers (Lcas215for/ Lcas462rev)**
100 K A	Low quality sequence (*Pseudomonas aeruginosa* 80% match)	*L. paracasei* 100%
100 K Ca	Low quality sequence (*Streptococcus thermophilus* 85.98% match)	*L. paracasei* 100%
100 K Co	*L. delbrueckii* subsp. *bulgaricus* 99% match	*L. paracasei* 100%
100 K H	*L. delbrueckii* subsp. *bulgaricus* 99% match	*L. paracasei* 99% match
100 K Y	*Pseudomonas gessardii* 99% match	*L. paracasei* 100% match

### 3.4. Tracking added *L. paracasei* BL23 EV in milk

#### 3.4.1. Size distribution

For this experiment, UHT skimmed milk from three different brands was purchased (coded as LA, PA, and PU), and the equivalent amount of EV isolated from *L. paracasei* BL23 grown in 200 mL of MRS was added to 200 mL of milk. Then, pellets from the successive centrifugations at 15, 33, and 100 K were collected in PBS and analyzed by DLS. In general, EVs of smaller size were recovered from centrifugations at slower speeds (Table 3), suggesting a higher EV density. In general terms, the size distribution of EV was very homogeneous within samples (Supplementary Figure 4), confirmed by the low standard deviations, particularly if compared to EV from the bacterial culture medium, possibly due to the low concentration of bacterial EV. Interestingly, peaks corresponding to possibly two different EV populations were recovered after ultracentrifugation at 100 K, the peak of smaller size EV, being on average 15–20 d.nm larger than the expected size of *L. paracasei* BL23 EV.

**Table 3:** EV size distribution, determined by DLS, of different milk samples with added *L. paracasei* EV recovered after 15, 33, and 100 K centrifugations.

	**EV form 15 K pellet**	**EV from 33 K pellet**	**EV from 100 K pellet**	
	**Pk 1 mean (d.nm; Sdev.)**	**Pk 1 mean (d.nm; Sdev.)**	**Pk 1 mean (d.nm; Sdev.)**	**Pk 2 mean (d.nm; Sdev.)**
LA	269.7 (11.5)	335.1 (2.2)	405.5 (35.42)	66.6 (19.0)
PU	188.4 (1.2)	183.6 (8.6)	302.3 (26.4)	57.9 (11.0)
PA	244.7 (15.1)	208.5 (9.3)	400.3 (89.7)	65.3 (18.6)
Total average	234.3 (37.3)	242.4 (70.7)	369.4 (70.9)	56.8 (25.7)

#### 3.4.2. Distribution of L. paracasei BL23 proteins P40 and P75 in the EV fractions

**Figure 3:**
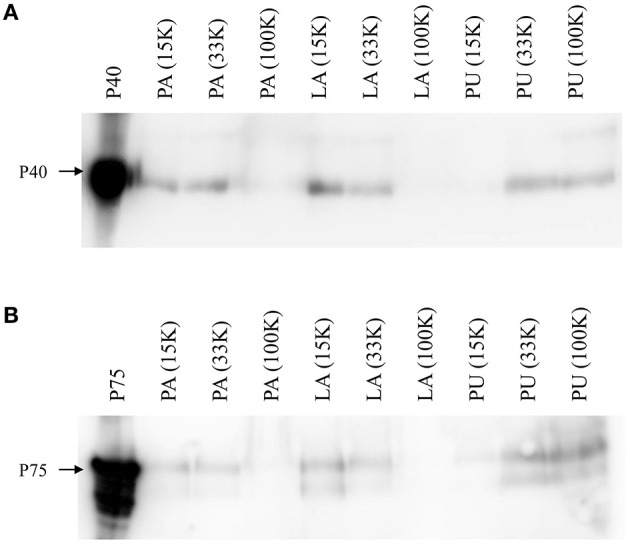
Detection of P40 **(A)** and P75 **(B)** by Western blot in EV isolated at different centrifugal pellets (15, 33, and 100 K), after the addition of *L. paracasei* EV to three different commercial UHT semi-skimmed milk products (PU, LA, and PU).

After the addition of EV from *L. paracasei* to commercial UHT milks, small EVs of the approximate size of *L. paracasei* EVs were detected in the 100 K pellets (see Section 3.4.1). The next step was the detection of P40 and P75 in EV prepared from the different milk pellets in Western blot analysis. Interestingly, both proteins were mainly found associated with 15 and 33 K fractions in two of the milk products (PA and LA; Figure 3). Milk PU had an unusual behavior when centrifuged at 100 K, and a very large pellet was collected at 100 K ultracentrifugation, indicating the presence of a polymeric gel. If the manufacturing process of this milk used a large proportion of powdered milk, it could contain stabilizers as adjuncts that were found in the sediment at high centrifugal speed. Consequently, the results obtained from this commercial milk may not be representative of what could be happening during natural fermentation.

#### 3.4.3. Presence of microbial DNA

As mentioned earlier, the presence of microbial DNA can be specifically associated with bacterial EV cargo. Hence, after the addition of *L. paracasei* EV to three different commercial UHT skimmed milk products (PU, LA, and PU), PCR detection of microbial 16S rDNA was performed on EV isolated at different centrifugal speeds (15, 33, and 100 K). Universal 16S rDNA primer amplicons obtained from all milk samples were sequenced. Most of the sequences were not “clean,” showing some background DNA sequences, and many of them had high homology to *Lactobacillus delbruekii* subsp. *bulgaricus* 16S rDNA, unknown bacteria, or were not quality sequences (Table 4). Then, 16S rDNA from *L. paracasei* was specifically amplified in all samples, and bands obtained were sequenced, indicating the presence of *L. paracasei* EV associated with all samples.

**Table 4:** Sequences obtained after the amplification of 16S rDNA from EV in the 100 K pellets of the different fermented dairy products.

**Milk samples with added BL23 EV**	**Universal bacterial 16S primers (27f-558r)**	***L. paracasei*** **specific 16S primers (Lcas215for/ Lcas462rev)**
100 K LA	Uncultured bacterium 95–96% match	*L. paracasei* 100% match
33 K LA	*L. delbrueckii* subsp. *bulgaricus* 99.19% match	*L. paracasei* 99% match
15 K LA	Not quality sequence (contaminated)	*L. paracasei* 99% match
100 K PA	Uncultured bacterium/*L. delbrueckii* subsp. *bulgaricus* 94% match	*L. paracasei* 99% match
33 K PA	*L. delbrueckii* subsp. *bulgaricus* 99,59% match	*L. paracasei* 100% match
15 K PA	*L. delbrueckii* subsp. *bulgaricus* 99% match	*L. paracasei* 99% match
100 K PU	• *L. delbrueckii* subsp. *bulgaricus* 98% match • Contaminant: 26 nt 100% match *Staphylococcus epidermidis*/ *haemolyticus/ saprophyticus*	*L. paracasei* 99% match
33 K PU	Uncultured bacterium 94% match	*L. paracasei* 100% match
15 K PU	*L. delbrueckii* subsp. *bulgaricus* 97% match (low quality sequence)	No significant similarity found (contaminated)

## 4. Discussion

The presence of EVs in all kingdoms of life suggests that they are present in food components; hence, higher organisms—including humans—would be regularly consuming large amounts of EVs of different natures. One of the main objectives of this study was to investigate the presence of *L. paracasei* EV in commercial fermented milk products. A previous article studied the presence of the anti-inflammatory and tissue-repairing proteins P40 and P75 in supernatants of some commercial fermented milk products prepared with *L. paracasei*, finding that P75 was always detected but not P40 (Bäuerl et al., 2019). Shortly after this, the same group showed that those proteins were adhered to the surface of EV from *L. paracasei* (Bäuerl et al., 2020). Some fermented dairy products contain probiotic strains of *L. paracasei;* therefore, it was interesting to know if *L. paracasei* could produce EV during milk fermentations, and what was the interaction between EV and P40 and P75 in commercial products. For that purpose, EVs were prepared from five different fermented drinks carrying *L. paracasei* strains, and the presence of P40 and P75 was investigated. Prior to further analysis, it was confirmed that commercial strains in the products produced EV, P40, and P75. EVs from fermented dairy products were prepared following conditions used from milk EVs, by successive centrifugations at 15, 33, and 100 K (Benmoussa et al., 2020). Some of the products rendered large lumpy pellets at lower speed centrifugations (15,000 × *g* and 33,000 × *g*), possibly due to the precipitation of milk proteins, as a consequence of the physicochemical changes occurring during the fermentation process. Given that the aim of this study was to find bacterial EV in fermented products, assays were initially focused on the 100 K centrifugal fractions and conditions used to recover EV from the bacterial culture supernatants. The particle size of EV from milk fermented products at 100 K had an average particle size by DLS of 147.26 d.nm (average *z*-value 120.95 d.nm), with no indication of the presence of small size *L. paracasei* EV (24.8–47.1 d.nm). This could be explained if the proportion of bacteria EV was small relative to milk EV, as large and more abundant EV may dissipate light more efficiently hampering the detection of smaller particles. In a preliminary assay, Western blot analysis of the 100 K pellets showed that a very small amount of P75 was present, and P40 was not detected. Therefore, pellets and supernatants from previous centrifugations were analyzed. It was previously shown that P75 can be attached to EV and also in solution, in contrast, P40 was always firmly attached to bacterial EV (Bäuerl et al., 2020). In agreement with this data, in fermented products, P75 was present in all pellets and supernatants, while P40 was detected only in the 33 K pellets and possibly 15 K pellets (faint signal) of two commercial products. These results suggested that both proteins may interact with membrane or perivesicular components of milk EV by electrostatic interactions, or alternatively, *L. paracasei* EV may be present in other centrifugal pellets when mixed with milk. In order to solve this question, a marker was required to detect bacterial EV in different pellets. LTA is a characteristic marker of the cell membranes of Firmicutes (Bäuerl et al., 2020), but here, the concentration of EV was below the sensitivity of the LTA antibodies used.

Empirical evidence showed that bacterial EV carry chromosomal DNA fragments (Biller et al., 2014; Bitto et al., 2017); in fact, new-generation sequencing techniques can be applied to study EV from bacterial gut populations and as health markers (Kameli et al., 2021; Park et al., 2021). In milk, the presence of indigenous nucleases (Stepaniak et al., 2003) in multifunctional proteins such as lactoferrin (Soboleva et al., 2019) could eliminate extravesicular DNA, for which PCR detection of bacterial 16S rDNA was implemented as a reliable cargo marker, and general bacterial 16S rDNA was used to detect bacterial EV. Total amplified bacterial DNA of 100 K pellets from fermented products rendered low-quality sequences where *L. paracasei* 16S rDNA was not found, but interestingly, sequences homologous to other bacterial 16S rDNAs were present (*L. delbrueckii* or *Pseudomonas* species). This indicated that these 100 K pellets contained a mixture of EV where *L. paracasei* EVs were underrepresented, but the technique could trace a previous microbiological history of the products, also observed in the EV from UHT milk. Then, specific *L. paracasei* primers were designed for the selective amplification of rDNA, and results showed that *L. paracasei* EVs were indeed present in all fermented products.

Since the concentration of *L. paracasei* EV was apparently very low in fermented products, large amounts of EV from *L. paracasei* BL23 were added to UHT milk in order to facilitate their detection in a milk environment and to test if P40 and P75 would bind to milk EV 15 and 33 K pellets, as suggested by the results from fermented products. After adding EV from *L. paracasei* to UHT milk samples, different fractions of EV were prepared by sequential centrifugations, and pellets were analyzed by DLS, Western blot, and 16S rDNA amplification and sequencing. UHT milk EVs have been reported to have large sizes (~200–230 nm) when determined by DLS (Benmoussa et al., 2017), which are normally larger than the sizes found by electron microscopy (Reinhardt et al., 2012), and this is possibly due to the presence of abundant perivesicular material (proteins and polysaccharides) that change the hydrodynamic diameter. In addition, UHT treatment structurally affects milk EV (Kleinjan et al., 2021). Vesicular properties cannot be extrapolated to the fermented products in this study because (heat) treatments used to prepare milk for the manufacture of commercial fermented milk could not be precisely determined from the product labels (Supplementary Table 1). Of note, it was not the scope of this article to study in detail UHT milk EVs because other fractionation methodologies should have been implemented (Kleinjan et al., 2021). The results showed that after adding *L. paracasei* BL23 EV to three UHT milk brands, the 100 K pellets showed dynamic light scattering populations of small EV (57.9–66.6 d.nm; Table 3), with a size similar to that found here for *L. paracasei* BL23 (41.9 ± 9.9 d.nm; Table 1). This, together with 16S rDNA analysis, supported the presence of *L. paracasei* EVs in the 100 K pellets, but P40 and P75 could not be found. As observed in the fermented products, P40 and P75 proteins were detected in EV from 15 and 33 K centrifugations. Bacterial DNA detection confirmed the presence of *L. paracasei* 16S rDNA in EV fractions obtained in all pellets, indicating that a fraction of *L. paracasei* EV was possibly isolated together with milk EV at 15 and 33 K centrifugations.

The general conclusions of this study underline that some of the commercial probiotics studied (A-BL413, H-BL417, and Y-BL418) produce P40 and P75 bound to EV at a high rate, much like reference probiotics such as *L. parcasei* BL23 or *L. rhamnosus* GG. In addition, different intensities of immunological signals for P40 and P75 in commercial fermented products could respond to fermentation parameters and environmental factors affecting the production of EV and functional proteins. Finally, other factors may possibly affect the functional benefits of P40 and P75 in these products, such as their likely association with milk EV. Most important is the fact that P40, and to a lesser extent P75, are not associated with native small-size bacterial EV, and they are probably complex to milk EV. Experimental data shown here would also support the aggregation or fusion of a fraction of *L. paracasei* EV with milk EV, including attached P40 and P75.

## Data availability statement

The original contributions presented in the study are included in the article/Supplementary material, further inquiries can be directed to gaspar.perez@iata.csic.es.

## Author contributions

KC-R and LG-P: experimental procedures and text editing. GP: planning, supervising, experimental procedures, and manuscript preparation. All authors contributed to the article and approved the submitted version.
