# Supplementary material for: Bacterial extracellular vesicles and associated functional proteins in fermented dairy products with Lacticaseibacillus paracasei
Source: Front Microbiol. 2023 Apr 20;14:1165202. doi: 10.3389/fmicb.2023.1165202 (PMC10157241; doi:10.3389/fmicb.2023.1165202)
Supplement: Supplementary file 1 [file Data_Sheet_1.pdf]

Supplementary Material

Bacterial Extracellular Vesicles and Associated Functional Proteins in Fermented Dairy Products with *Lacticaseibacillus paracasei*

Gaspar Pérez Martínez*, Lola Giner-Pérez, Keshia F. Castillo-Romero

*** Correspondence:**
Gaspar Pérez Martínez

gaspar.perez@iata.csic.es

Table S1.- List of commercial products used in this study containing strains of *L. paracasei*, their concentration in the product (cfu/ml) and taxonomic identification by 16S rDNA sequencing.

**Product**	**Source of milk in ingredients**	**pH**	**Colony Counts (cfu/ml)**	**Identification of isolated strain (16S rDNA)**	**Strain code**
A	Pasteurized skim milk and skim milk powder	4.50	5.87×10^8	*Lacticaseibacillus paracasei*	BL413
Ca	Pasteurized skim milk and skim milk powder	4.65	13.2×10^8	*Lacticaseibacillus paracasei*	BL415
H	Pasteurized semi-skimed milk	4.53	7.8×10^8	*Lacticaseibacillus paracasei*	BL416
Co	Semi-skimed milk	4.39	7.5×10^8	*Lacticaseibacillus paracasei*	BL417
Y	Skim milk powder	3.80	4.13×10^8	*Lacticaseibacillus paracasei*	BL418

Figure S1.- Sequences of the satellite region in *cmu*B gene (encoding for P75) containing tandem repeats, in the *L. paracasei* strains isolated from commercial products.

For Ethic Issues Compliance: sequences of the 16S rDNA fragments amplified from the isolated strains had an identity of 99-100% to those previously reported for *Lacticaseibacillus paracasei*, *Lactobacillus delbrueckii subsp. bulgaricus* and *Pseudomonas gessardii*. In other cases, the low sequence quality question their real value, but they are mentioned to state the likely presence of diverse 16S rDNAs.

Figure S2.- DLS spectra with size distribution (ordinate) by light scattering intensity (abscissa) of repeated measurements of EV recovered from 100K centrifugations of MRS culture supernatants of probiotic strains isolated from different commercial products

Figure S3.- DLS spectra of the size distributions in samples recovered after 100K centrifugation of the different fermented dairy products.

Figure S4.- DLS spectra of the size distributions in samples recovered after 15K, 33K and 100K centrifugations of the different milk samples with added *L. paracasei* EV.

Figure S1

```
BL23    AGTGCTGCCGCAGAATCCAGTGCTGCTGCAGAGTCCAGTGCTGCCGCATC
Ca      AGTGCTGCCGCAGTTCCAAGTGCTGCTGCAGATCTAAGTGCTGCTGCATC
A       AGTGCTGCCGCAGAATCCAGTGCTGCTGCAGAGTCCAGTGCTGCCGCATC
H       AGTGCTGCCGCAGAATCCAGTGCTGCTGCAGAGTCCAGTGCTGCCGCATC
Co      AGTGCTGCCGCAGAATCCAGTGCTGCTGCAGAGTCCAGTGCTGCCGCATC
Y       AGTGCTGCCGCAGAATCCAGTGCTGCTGCAGAGTCCAGTGCTGCCGCATC

BL23    GAAGGCTGCTGCTGATTCTAGCGCTGCAGCTGTGCAGACTACGACGCCTG
Ca      AAAGGCTGCTGCTGATTCTAGCGCTGCAGCTGTGCAGACTACGACGCCTG
A       GAAGGCTGCTGCTGATTCTAGCGCTGCAGCTGTGCAGACTACGACGCCTG
H       GAAGGCTGCTGCTGATTCTAGCGCTGCAGCTGTGCAGACTACGACGCCTG
Co      GAAGGCTGCTGCTGATTCTAGCGCTGCAGCTGTGCAGACTACGACGCCTG
Y       GAAGGCTGCTGCTGATTCTAGCGCTGCAGCTGTGCAGACTACGACGCCTG

BL23    AGTCCAGTGCTGCGCCTGCAACGACACAGGTTGATGCAACTCAGGAACAG
Ca      AGTCCAGTGCTGCGCCTGCAACGACACAGGTTGATGCAACTCAGGAACAG
A       AGTCCAGTGCTGCGCCTGCAACGACACAGGTTGATGCAACTCAGGAACAG
H       AGTCCAGTGCTGCGCCTGCAACGACACAGGTTGATGCAACTCAGGAACAG
Co      AGTCCAGTGCTGCGCCTGCAACGACACAGGTTGATGCAACTCAGGAACAG
Y       AGTCCAGTGCTGCGCCTGCAACGACACAGGTTGATGCAACTCAGGAACAG

BL23    CAACAGCAGGCAGAACCAAGCAATACGGTCAATACCGAGGAAACGACAAA
Ca      CAACAGCAGGCAGAACCAAGCAATACGGTCAATGCCGAGGAAACGACAAA
A       CAACAGCAGGCAGAACCAAGCAATACGGTCAATACCGAGGAAACGACAAA
H       CAACAGCAGGCAGAACCAAGCAATACGGTCAATACCGAGGAAACGACAAA
Co      CAACAGCAGGCAGAACCAAGCAATACGGTCAATACCGAGGAAACGACAAA
Y       CAACAGCAGGCAGAACCAAGCAATACGGTCAATACCGAGGAAACGACAAA

BL23    TAACGCGACGCCGACTCCTGCACCAACGCCGACGCCTGCACCAACCCCAG
Ca      TAACGCG------------------------ACGCCTGCACCAACCCCAG
A       TAACGCGACGCCGACTCCTGCACCAACGCCGACGCCTGCACCAACCCCAG
H       TAACGCGACGCCGACTCCTGCACCAACGCCGACGCCTGCACCAACCCCAG
Co      TAACGCGACGCCGACTCCTGCACCAACGCCGACTCCTGCACCAACCCCAG
Y       TAACGCGACGCCGACTCCTGCACCAACGCCGACGCCTGCACCAACCCCAG
```

Figure S2

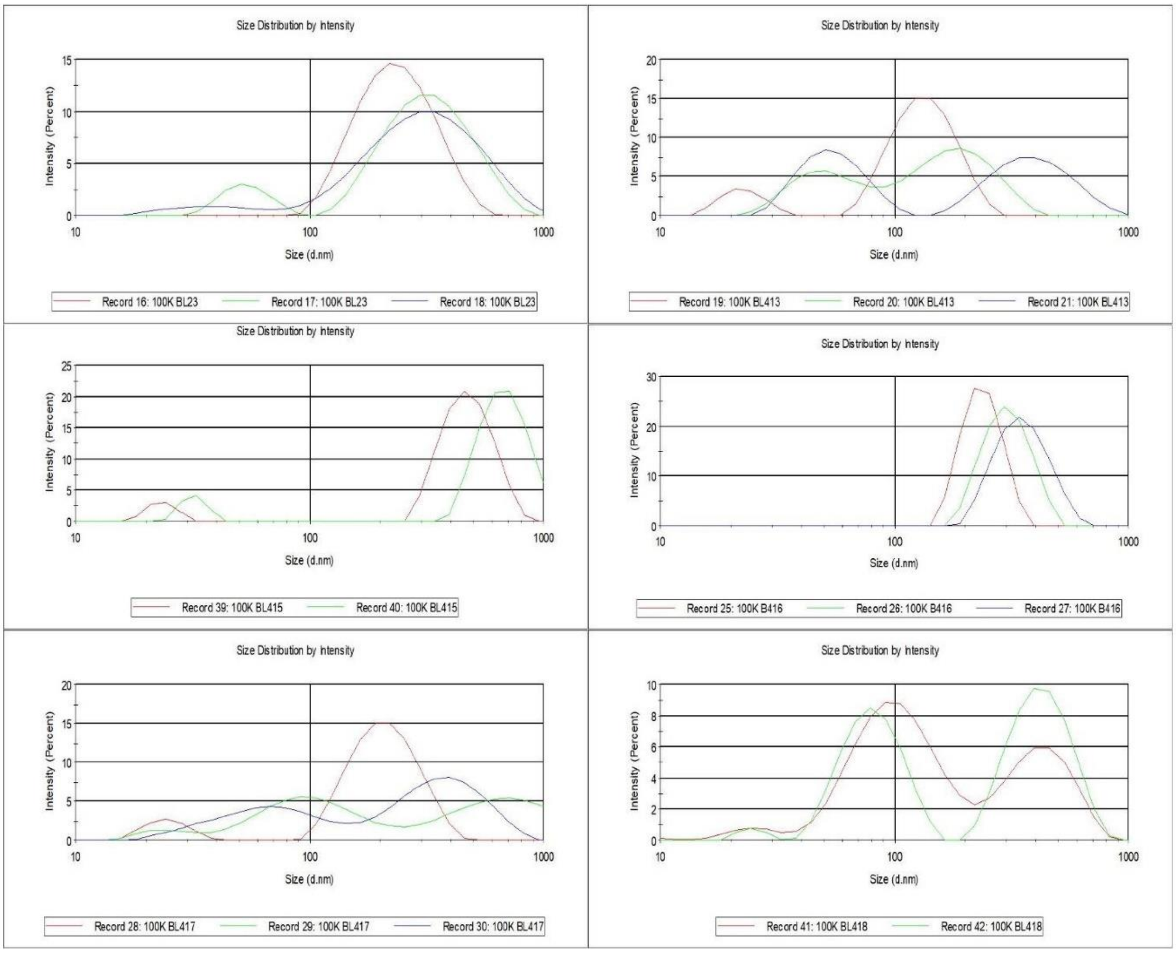
Size Distribution by Intensity
Intensity (Percent)
Size (d.nm)
Record 16: 100K BL23
Record 17: 100K BL23
Record 18: 100K BL23
Size Distribution by Intensity
Intensity (Percent)
Size (d.nm)
Record 19: 100K BL413
Record 20: 100K BL413
Record 21: 100K BL413
Size Distribution by Intensity
Intensity (Percent)
Size (d.nm)
Record 39: 100K BL415
Record 40: 100K BL415
Size Distribution by Intensity
Intensity (Percent)
Size (d.nm)
Record 25: 100K B416
Record 26: 100K B416
Record 27: 100K B416
Size Distribution by Intensity
Intensity (Percent)
Size (d.nm)
Record 28: 100K BL417
Record 29: 100K BL417
Record 30: 100K BL417
Size Distribution by Intensity
Intensity (Percent)
Size (d.nm)
Record 41: 100K BL418
Record 42: 100K BL418

Figure S3

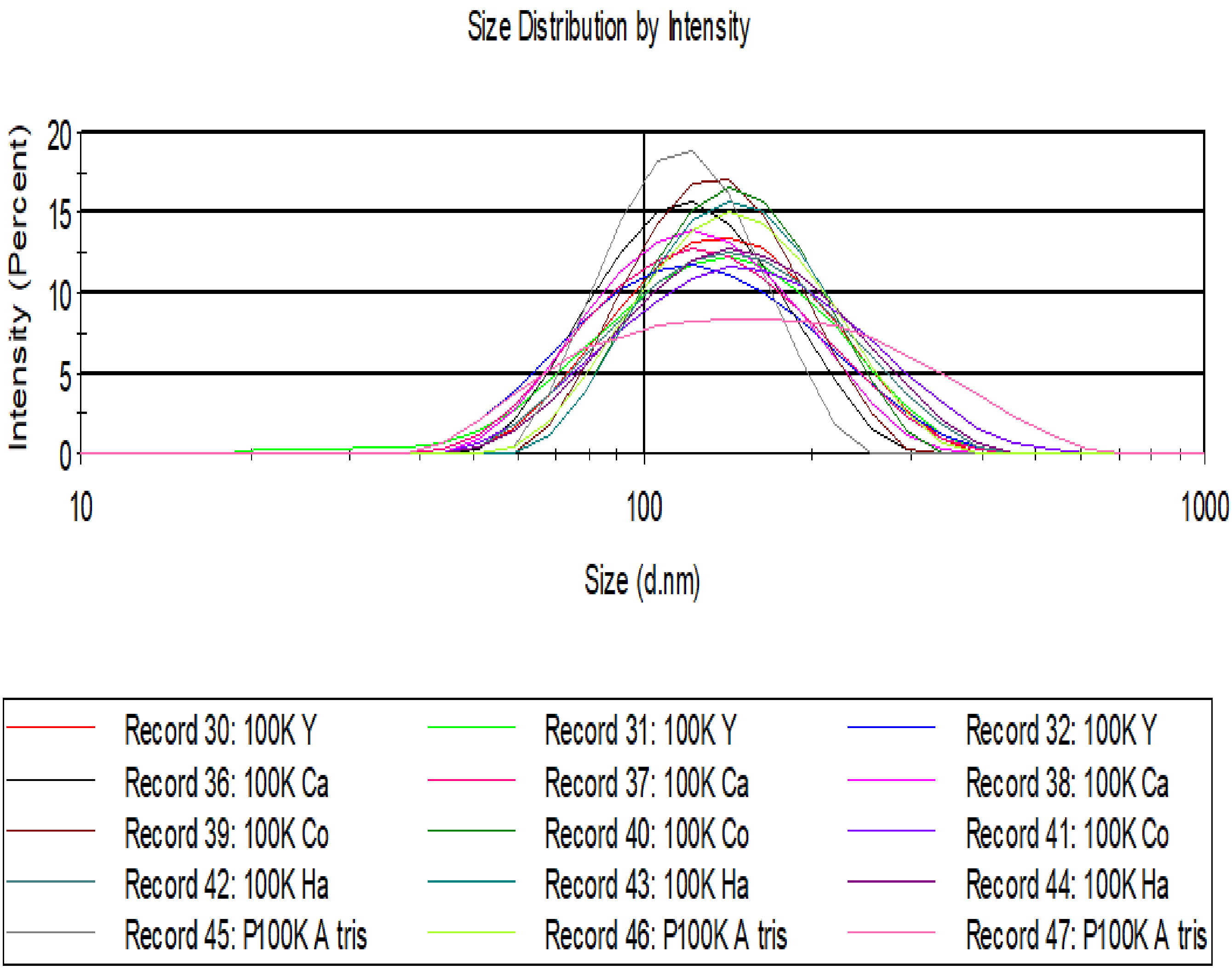
Size Distribution by Intensity
Intensity (Percent)
20
15
10
5
0
10
100
1000
Size (d.nm)
Record 30: 100K Y
Record 31: 100K Y
Record 32: 100K Y
Record 36: 100K Ca
Record 37: 100K Ca
Record 38: 100K Ca
Record 39: 100K Co
Record 40: 100K Co
Record 41: 100K Co
Record 42: 100K Ha
Record 43: 100K Ha
Record 44: 100K Ha
Record 45: P100K A tris
Record 46: P100K A tris
Record 47: P100K A tris

Figure S4

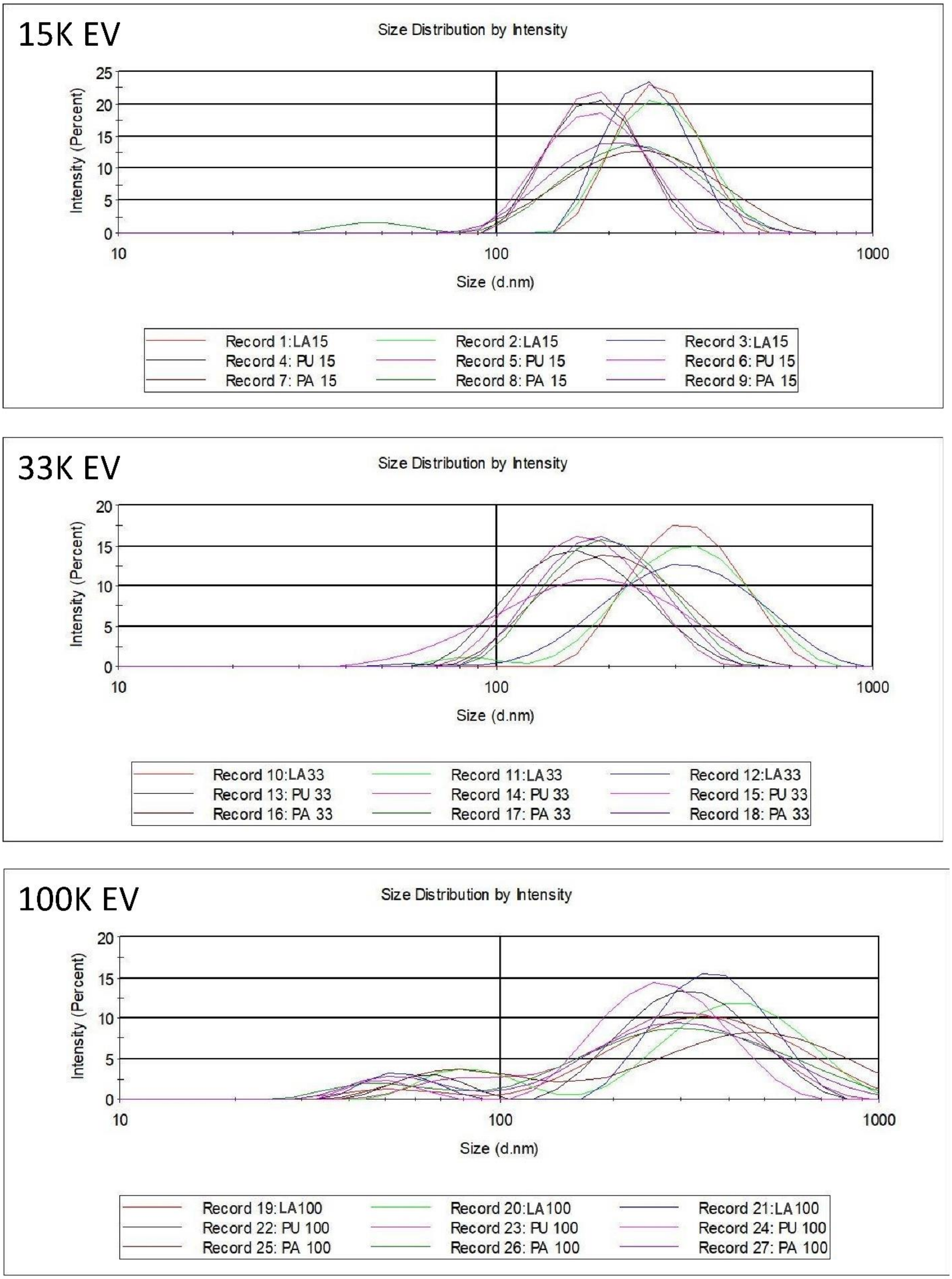
15K EV
Size Distribution by Intensity
Intensity (Percent)
Size (d.nm)
Record 1:LA15
Record 2:LA15
Record 3:LA15
Record 4: PU 15
Record 5: PU 15
Record 6: PU 15
Record 7: PA 15
Record 8: PA 15
Record 9: PA 15
33K EV
Size Distribution by Intensity
Intensity (Percent)
Size (d.nm)
Record 10:LA33
Record 11:LA33
Record 12:LA33
Record 13: PU 33
Record 14: PU 33
Record 15: PU 33
Record 16: PA 33
Record 17: PA 33
Record 18: PA 33
100K EV
Size Distribution by Intensity
Intensity (Percent)
Size (d.nm)
Record 19:LA100
Record 20:LA100
Record 21:LA100
Record 22: PU 100
Record 23: PU 100
Record 24: PU 100
Record 25: PA 100
Record 26: PA 100
Record 27: PA 100
